# A Cross-Sectional Study of Psychological Status in Different Epidemic Areas in China After the COVID-19 Outbreak

**DOI:** 10.3389/fpsyt.2020.575705

**Published:** 2020-11-05

**Authors:** Huan Cao, Chengchao Zuo, Guo Li, Yaqi Huang, Ling Li, Shu Huang, Jianling Zhao, Jingjing Liu, Yongsheng Jiang, Furong Wang

**Affiliations:** ^1^Department of Neurology, Tongji Hospital, Tongji Medical College, Huazhong University of Science and Technology, Wuhan, China; ^2^Cancer Center of Tongji Hospital, Tongji Medical College, Huazhong University of Science and Technology, Wuhan, China

**Keywords:** COVID-19, depression, anxiety, psychological stress, health care workers

## Abstract

**Background:** The outbreak of coronavirus disease 2019 in Wuhan, Hubei Province, China has seriously affected people's mental health. We aimed to assess the psychological impact of the coronavirus disease 2019 on health care workers and non-health care workers in three different epidemic areas in China and to identify independent risk factors.

**Methods:** We surveyed 1,020 non-health care workers and 480 health care workers in Wuhan, other cities in Hubei except Wuhan and other provinces in China except Hubei.

**Results:** Health care workers in Hubei had higher levels of anxiety and depression than non-health care workers (*p* < 0.05), but there was no such difference in other provinces in China except Hubei (*p* > 0.05). Compared with other regions, health care workers in Wuhan was more anxious (*p* < 0.05), and this anxiety may be caused by concerns about occupational exposure and wearing protective clothing for a long time daily; health care workers in Hubei had more obvious depression (*p* < 0.05), which may be associated with long days participating in epidemic work and wearing protective clothing for a long time daily. Meanwhile, 62.5% of health care workers were proud of their work. The anxiety and depression of non-health care workers in Wuhan were also the most serious.

**Conclusions:** In Wuhan, where the epidemic is most severe, levels of anxiety and depression seem to be higher, especially among health care workers. This information may help to better prepare for future events.

## Introduction

In December 2019, a new type of coronavirus disease 2019 (COVID-19) appeared in Wuhan and spread rapidly domestically and internationally, posing a global health emergency. As of March 7, 2020, more than 80,000 people in China have been diagnosed with COVID-19, and more than 100,000 people worldwide have been diagnosed. Confirmed cases have been reported in all provinces of China. The growing number of confirmed and suspected cases, as well as the geographical spread of the disease, has raised public concern about infection, triggering public panic and psychological stress (1, 2).

Studies have shown that the public exhibited significant mental disorders such as fear, stress, sleep disorders, anxiety, and depression during the COVID-19 epidemic (3). Based on experiences of severe acute respiratory syndrome (SARS), it was suggested that these psychological problems were closely related to the severity of the epidemic (4). The stress effects of infectious diseases may be qualitatively distinct from those of other disasters, especially for health care workers (HCWs) (5). HCWs, overworked at the front line, have a high risk of occupational exposure and infection (6). Infection rates among HCWs in various countries during SARS ranged from 3 to 51% (7). As of February 17, 2020, Chinese officials reported that more than 3,000 HCWs had been infected with COVID-19. Another important reason for the psychological burden on HCWs is the greater fear of their family members being at a higher risk of infection (8–11). Therefore, when new epidemics break out, HCWs often suffer from huge psychological health problem, such as anxiety and depression (12, 13).

The psychological disorder is not only a temporary process and it may persist for a few years after the epidemic ends (14). Therefore, in addition to identify pathogenic factors, pathophysiology, clinical manifestations, diagnosis and treatment, it is also important to study the impact of COVID-19 outbreak on people's mental health. Understanding the psychological impact during an outbreak, such as anxiety levels, can not only help predict key behavioral outcomes (e.g., wearing a face-mask) (4), but may also have important implications for future psychological and behavioral research and public health interventions related to respiratory communicable diseases. Although there has been some research in this area, we have also studied the psychological conditions of the general public and HCWs in three different epidemic areas in China during the outbreak period. Specifically, this study investigated the levels of anxiety and depression of HCWs and non-HCWs, determined the impact of the severity of the epidemic on people in corresponding regions (The epidemic is worst in Wuhan, followed by other cities in Hubei except Wuhan, and the situation in other provinces in China except Hubei are better than these two regions), and evaluated the work perceptions of HCWs.

## Methods

### Participants

People aged 18 or above from all walks of life in Wuhan, other cities in Hubei except Wuhan, and other provinces in China except Hubei were welcome to join in the survey. Other cities in Hubei except Wuhan include 12 cities, autonomous prefecture in Hubei. Other provinces in China except Hubei include 30 provinces, autonomous regions and municipalities in the mainland of China. The classification was based on their geographic location, which was also closely related to the severity of the epidemic (e.g., Wuhan, other regions inside Hubei Province, and regions outside Hubei Province). The sample size was calculated to be 1,568. All participants are divided into HCWs (i.e., doctors and nurses) and non-HCWs (Others except doctors and nurses). Sociodemographic characteristics including name, gender, age, education level, occupation, annual family income, type of medical insurance, household registration location, current city.

### Data Collection

The questionnaire was collected through social media WeChat (15) (Tencent, Shenzhen, China) using an online survey platform Sojump (Changsha ran Xing InfoTech Ltd.,) during February 6th to 13th, 2020, when the epidemic was in the outbreak period, the situation was chaotic (16–18) and the number of people infected with COVID-19 was escalating. The convenience sampling was adopted. After the researchers explained the purpose of the study, 1,568 volunteers completed the questionnaire-based surveys, of which 1,500 were valid. Among the three regions, there were 1,020 questionnaires for non-HCWs and 480 questionnaires for HCWs. Each person can only answer one questionnaire. Sojump APP can set the number of answers, which can be set as each WeChat account is only allowed to answer once, and each IP is only allowed to answer once. Informed consent is exempt. Ethical approval was received from the Institutional Review Board (IRB) of the University of Huazhong university of science and technology. The ethical number is TJ-IRB20200322.

#### Basic Information Survey

A self-designed questionnaire was used to determine the relevant factors. Basic information about COVID-19 including the degree of attention to the COVID-19, whether relatives or friends have the COVID-19, and the current status (healthy home quarantine, suspected isolation, mild illness isolation, fever outpatient service, hospitalization, square cabin hospital). For HCWs, we added some additional items that can reflect their occupational exposure risk, work intensity, worries and current thoughts about their occupation, including operating post (fever outpatient service, inpatient department, square cabin hospital, others), the number of days participating in epidemic work, rest days in the past month, the average working hours per week, daily wear protective clothing time (hours), whether you are worried about occupational exposure (none, occasionally, often), whether you are worried about your family's cross-infection because of you, and the main feelings about your career (pride, fear, pessimism, it doesn't matter).

#### Anxiety and Depression Status Assessment

The assessment scale was composed of the Hamilton Anxiety Rating Scale (HAMA; Hamilton, 1959) and Hamilton Depression Rating Scale (HAMD; Hamilton, 1960), both of which had high reliability reported in the literature (the reliability of HAMA ranged between 0.82 and 0.94; the reliability of HAMD ranged between 0.81 and 0.98) (19–21). In this study, HAMA and HAMD also showed good internal consistency, with Cronbach's α coefficients of the total scale being 0.94 and 0.90 respectively. HAMA includes 14 items, each with 0~4 points, corresponding to asymptomatic, mild, moderate, severe, and extremely severe. Total score ≥ 29 points, there may be severe anxiety; Total score 21~28 points, there must be significant anxiety; Total score 14~20 points, there must be anxiety; Total score 7~13 points, there may be anxiety; Total score < 7 points, no anxiety symptoms. HAMD includes 24 items, a score of 0 in each item represents no symptoms. Total score > 35: major depression; Total score 21~35: definitely having depression; Total score 8~20: possible depression; Total score < 8, normal.

### Statistical Analysis

All statistical analyses were conducted using SPSS 19. The measurement data are expressed as mean ± standard deviation, independent sample *t*-test was used for comparison between the two groups. The counting data were represented by the number of cases (%), and the comparison was tested using χ^2^. Finally, the risk of anxiety and depression was further analyzed by multivariate logistic regression (using likelihood ratio estimation). Two-sided test with *p* < 0.05 was considered statistically significant.

## Results

### Sociodemographic Characteristics

Table 1 shows the main demographic characteristics of the participants and their scores on measures of anxiety and depression. A total of 1,568 questionnaires were collected, of which 1,500 were valid and the effective rate was 95.66%. Among them, female accounted for 82.08% of HCWs and 61.57% of non-HCWs. The mean age of HCWs and non-HCWs were 35.29 ± 8.85 years and 31.87 ± 11.25 years, respectively. The education levels of HCWs were Bachelor degree or above (82.92%) and Junior college or technical secondary school (17.08%). The education level of non-HCWs were Bachelor degree or above (43.73%) and Junior college or technical secondary school (51.37%), the other 4.9% including primary, middle and high school. The average HAMA scores for HCWs and non-HCWs were 4.30 ± 5.95 and 3.29 ± 5.92, and the average HAMD scores for HCWs and non-HCWs were 8.19 ± 7.97 and 6.35 ± 7.93.

**Table 1:** Sociodemographic characteristics.

	**HCWs**	**non-HCWs**	***p***
Sex, *n* (%)			<0.05
Female	394(82.08)	628(61.57)	
Male	86(17.92)	392(38.43)	
Age	35.29 ± 8.85	31.87 ± 11.25	<0.05
Education, *n* (%)			<0.05
Bachelor degree or above	398(82.92)	446(43.73)	
Junior college or technical secondary	82(17.08)	524(51.37)	
school			
HAMA	4.30 ± 5.95	3.29 ± 5.92	<0.05
HAMD	8.19 ± 7.97	6.35 ± 7.93	<0.05

### Anxiety

In general, HCWs were more anxious than non-HCWs (χ^2^ = 9.36, *p* < 0.05, Table 2). HAMA score less than 7 is considered to have no anxiety, accounting for 75.83% in HCWs and 82.55% in non-HCWs. Similarly, in Wuhan (χ^2^ = 7.32, *p* < 0.05) and other cities in Hubei except Wuhan (χ^2^ = 6.40, *p* < 0.05), HCWs was also more anxious than non-HCWs (Table 2). But in other provinces in China except Hubei, there was no significant difference in anxiety levels between HCWs and non-HCWs (χ^2^ = 0.81, *p* > 0.05, Table 2).

**Table 2:** Comparison of anxiety among HCWs and non-HCWs.

	***N***	**Composition of HAMA**				
		***n*****(%)**				
		**<7**	**7**~**13**	**>14**	****χ**^2^**	***p***
All samples	1,500	1206(80.4)	199(13.27)	95(6.33)		
HCWs	480	364(75.83)	78(16.25)	38(7.92)		
non-HCWs	1,020	842(82.55)	121(11.86)	57(5.59)	9.36	<0.05
**Wuhan**						
HCWs	134	83(61.94)	32(23.88)	19(14.18)		
non-HCWs	208	157(75.48)	34(16.35)	17(8.17)	7.32	<0.05
**Other cities in Hubei except Wuhan**						
HCWs	207	161(77.78)	33(15.94)	13(6.28)		
non-HCWs	361	310(85.87)	34(9.42)	17(4.71)	6.40	<0.05
**Other provinces in China except Hubei**						
HCWs	139	120(86.33)	13(9.35)	6(4.32)		
non-HCWs	451	375(83.15)	53(11.75)	23(5.10)	0.81	>0.05

The anxiety levels of HCWs in other cities in Hubei except Wuhan (χ^2^ = 10.95, *p* < 0.05) and other provinces in China except Hubei (χ^2^ = 21.44, *p* < 0.05) were significantly lower than that in Wuhan, but there was no significant difference in HCWs anxiety levels between other cities in Hubei except Wuhan and other provinces in China except Hubei (χ^2^ = 4.05, *p* > 0.05, Table 3). Different from HCWs, although the anxiety level of non-HCWs in Wuhan was higher than that in other cities in Hubei except Wuhan (χ^2^ = 9.69, *p* < 0.05), it was not significantly higher than that in other provinces in China except Hubei (χ^2^ = 5.53, *p* > 0.05). Moreover, there was no significant difference between other cities in Hubei except Wuhan and other provinces in China except Hubei (χ^2^ = 1.26, *p* > 0.05, Table 3).

**Table 3:** Comparison of anxiety in different areas.

	***N***	**Composition of HAMA**						
		***n*****(%)**						
		**<7**	**7**~**13**	**>14**	****χ**^2^**	P*	****χ**^2^**	***P#***
**HCWs**								
Wuhan	134	83(61.94)	32(23.88)	19(14.18)				
Other cities in Hubei except Wuhan	207	161(77.78)	33(15.94)	13(6.28)	10.95	<0.05*		
Other provinces in China except Hubei	139	120(86.33)	13(9.35)	6(4.32)	21.44	<0.05*	4.05	>0.05
**non-HCWs**								
Wuhan	208	157(75.48)	34(16.35)	17(8.17)				
Other cities in Hubei except Wuhan	361	310(85.87)	34(9.42)	17(4.71)	9.69	<0.05*		
Other provinces in China except Hubei	451	375(83.15)	53(11.75)	23(5.10)	5.53	>0.05	1.26	>0.05

P* compared with Wuhan, P# comparison between other cities in Hubei except Wuhan and other provinces in China except Hubei.

Next, we explored the reasons for the different anxiety levels of HCWs in different regions. We used the multivariable logistic regression analysis to test the variables of sex, age, whether you often worry about occupational exposure, whether you are worried about your family's cross-infection because of you, daily wearing protective clothing time (hours) and participation in epidemic work(days). It was found that whether you often worry about occupational exposure (OR 2.833; 95% CI 1.274–6.298) and daily wearing protective clothing time (hours) (OR 1.086; 95% CI 1.034–1.140) were the independent risk factors (<0.05) (Table 4).

**Table 4:** Risk of anxiety: logistic regression analysis.

**Variable**	**OR (95% CI)**	***p***
Male	1.254 (0.455–3.460)	0.662
Age	1.006 (0.964–1.050)	0.784
Whether you often worry about occupational exposure (yes)	2.833 (1.274–6.298)	0.011
Whether you are worried about your family's cross-infection because of you (no)	0.272 (0.035–2.116)	0.214
Daily wear protective clothing time (hours)	1.086 (1.034–1.140)	0.001
Participation in epidemic work(days)	1.012 (0.985–1.039)	0.397

*OR, odds ratio; CI, confidence interval*.

### Depression

Compared with non-HCWs, HCWs showed significant depression (χ^2^ = 36.03, *p* < 0.05, Table 5). Similar to the anxiety condition, the depression levels of HCWs were more serious than that of non-HCWs in Wuhan (χ^2^ = 9.67, *p* < 0.05) and other cities in Hubei except Wuhan (χ^2^ = 30.52, *p* < 0.05). There was no significant difference between other cities in Hubei except Wuhan and other provinces in China except Hubei (χ^2^ = 0.56, *p* > 0.05, Table 5).

**Table 5:** Comparison of depression among HCWs and non-HCWs.

		**Composition of HAMD**				****χ**^2^**	***p***
		***n*****(%)**					
	***N***	**<8**	**8**~**20**	**21**~**35**	**>35**		
All samples	1500	1036(69.07)	343(22.87)	103(6.87)	18(1.20)		
HCWs	480	288(60.00)	142(29.58)	48(10.00)	2(0.42)		
non-HCWs	1020	748(73.33)	201(19.71)	55(5.39)	16(1.57)	36.03	<0.05
**Wuhan**							
HCWs	134	65(48.51)	45(33.58)	24(17.91)	0(0.00)		
non-HCWs	208	125(60.10)	56(26.92)	22(10.58)	5(2.40)	9.67	<0.05
**Other cities in Hubei except Wuhan**							
HCWs	207	119(57.49)	68(32.85)	19(9.18)	1(0.48)		
non-HCWs	361	282(78.12)	59(16.34)	15(4.16)	5(1.39)	30.52	<0.05
**Other provinces in China except Hubei**							
HCWs	139	104(74.82)	29(20.86)	5(3.60)	1(0.72)		
non-HCWs	451	341(75.61)	86(19.07)	18(3.99)	6(1.33)	0.56	>0.05

Further analysis showed that, among the three regions, HCWs in Wuhan (χ^2^ = 25.82, *p* < 0.05) and other cities in Hubei except Wuhan (χ^2^ = 11.95, *p* < 0.05) had significant differences in depression levels compared with those in other provinces in China except Hubei (Table 6). Unlike anxiety, non-HCWs in other cities in Hubei except Wuhan (χ^2^ = 22.45, *p* < 0.05) and other provinces in China except Hubei (χ^2^ = 20.07, *p* < 0.05) had lower depression levels than those in Wuhan. There was no difference between other cities in Hubei except Wuhan and other provinces in China except Hubei (χ^2^ = 1.02, *p* > 0.05, Table 6).

**Table 6:** Comparison of depression in different areas.

		**Composition of HAMD**				****χ**^2^**	p*	****χ**^2^**	***p*#**
		***n*****(%)**							
	***N***	**<8**	**8**~**20**	**21**~**35**	**>35**				
**HCWs**									
Wuhan	134	65(48.51)	45(33.58)	24(17.91)	0(0.00)				
Other cities in Hubei except Wuhan	207	119(57.49)	68(32.85)	19(9.18)	1(0.48)	6.79	>0.05		
Other provinces in China except Hubei	139	104(74.82)	29(20.86)	5(3.60)	1(0.72)	25.82	<0.05*	11.95	<0.05#
**non-HCWs**									
Wuhan	208	125(60.10)	56(26.92)	22(10.58)	5(2.40)				
Other cities in Hubei except Wuhan	361	282(78.12)	59(16.34)	15(4.16)	5(1.39)	22.45	<0.05*		
Other provinces in China except Hubei	451	341(75.61)	86(19.07)	18(3.99)	6(1.33)	20.07	<0.05*	1.02	>0.05

P* compared with Wuhan, P# comparison between other cities in Hubei except Wuhan and other provinces in China except Hubei.

Finally, we explored the causes of different levels of depression in HCWs in different regions. It was found that daily wearing protective clothing time (hours) (OR 1.100; 95% CI 1.040–1.163) and participation in epidemic work(days) (OR 1.030; 95% CI 1.006–1.054) were the independent risk factors (Table 7).

**Table 7:** Risk of depression: logistic regression analysis.

**Variable**	**OR (95% CI)**	***p***
Male	1.433 (0.563–3.651)	0.450
Age	0.996 (0.957–1.036)	0.826
Whether you often worry about occupational exposure (yes)	1.587 (0.816–3.086)	0.173
Whether you are worried about your family's cross-infection because of you (no)	0.350 (0.079–1.543)	0.165
Daily wear protective clothing time (hours)	1.100 (1.040–1.163)	0.001
Participation in epidemic work(days)	1.030 (1.006–1.054)	0.012

*OR, odds ratio; CI, confidence interval*.

## Discussion

At the end of 2019, the outbreak of COVID-19 swept China and affected the whole world. Due to the severe situation of the epidemic, it was listed as a public health emergency of international concern (PHEIC) by WHO on January 30, 2020 (22). In China, people's normal work and life were interrupted, all residents were quarantined at home, the situation was chaotic and China was pressed the pause button. There was a widespread feeling of perceived life threat, extreme vulnerability, uncertainty and helplessness at that time. This fear spread within the public and also the hospitals, causing panic. As with any disaster, the influence and trauma of COVID-19 outbreak will lead to anxiety and depression, the overall levels of anxiety and depression obtained in this study were similar to the results of other studies (23, 24). Furthermore, we found that HCWs in Wuhan and other cities in Hubei except Wuhan had higher levels of anxiety and depression than non-HCWs, but there was no such difference in other provinces in China except Hubei. Compared with the other two regions, the HCWs in Wuhan was more anxious, and this anxiety might be caused by concerns about occupational exposure and wearing protective clothing for a long time daily. Compared with other provinces in China except Hubei, HCWs in the remaining two regions had more obvious depression, which might be associated with long days participating in epidemic work and wearing protective clothing for a long time daily. Meanwhile, 62.5% of HCWs were proud of their work. The anxiety and depression of non-HCWs in Wuhan were also the most serious.

We assessed both anxiety and depression levels in HCWs and non-HCWs. The results suggest that HCWs are worse off than non-HCWs in both anxiety and depression. This is not hard to predict, as the burden on HCWs is greater, they faced high risk of infection, insufficient contamination protection, overwork, negative emotions from patient, exhaustion, discrimination, isolation, etc. (18, 25, 26). A literature review concluded that rescue workers have a higher incidence of post-traumatic stress disorder (PTSD) than the general population (27). In the early stages of the epidemic, the HCWs faced the uncertainty of encountering an undiagnosed COVID-19 patient every day, and there was no awareness of the need for systemic protection. When the epidemic broke out in full, the HCWs worked at the front line, and medical staff and resources were insufficient, their work intensity was high (28). As a result, both the occupational exposure and the risk of infection have greatly increased. Coupled with the fear of cross-infection among family members due to them, their psychological burden is heavier and pressure is greater. In addition, the differences in anxiety and depression levels between HCWs and non-HCWs in the three regions also implies that HCWs in Hubei are under more pressure and that the epidemic situation in Hubei is more serious which is consistent with the reality. The existing research has rarely investigated psychological status of HCWs and non-HCWs in Wuhan, other regions inside Hubei Province and regions outside Hubei Province during the COVID-19 outbreak (5, 12).

In order to clarify whether there is a difference in the psychological state of HCWs in different epidemic regions, we discussed the anxiety and depression of HCWs in Wuhan, other cities in Hubei except Wuhan and other provinces in China except Hubei. Our results suggest that during the outbreak of COVID-19 in mainland China, HCWs in high risk areas may have a higher prevalence of anxiety and depression. A study involving 34 hospitals showed that health care workers in Wuhan had more serious psychological problems (12). Other studies had also found that the proportion of frontline medical workers with anxiety and depression was significantly higher (29, 30). A recent comparative study showed that, at a time when confirmed COVID-19 cases rose sharply in Hong Kong and the epidemic in Hubei was under control, the prevalence of depression among health workers in Hong Kong (50.4%) were more severe than in Hubei province (15.1%) (25). It should be noted that the mental problems of both HCWs and general population in Hong Kong were reported to be exceptionally high (31). Since the performance of depressive symptoms is affected by comprehensive and complex internal and external factors, this discrepancy may due to the differences of regions, time points of the research, evaluation scales or sample sizes. Study on SARS have also shown that high-risk HCWs experience fatigue and poor sleep, worrying about health, accompanied by increased depression, anxiety, and post-traumatic stress scores (32). Moreover, we found that non-HCWs in Wuhan had a higher level of severe anxiety than other cities in Hubei except Wuhan, and a higher proportion of depression than the remaining two regions. In general, anxiety and depression levels among HCWs and non-HCWs in Wuhan, where the epidemic was most severe, were higher than elsewhere. Our conclusions were supported by a longitudinal study, which found that during SARS, anxiety levels were strongly correlated with the severity of the epidemic and closely mirrored the number of new cases per day (4, 25, 33, 34).

Then, we assessed the perception of job among HCWs during the COVID-19 epidemic and the risk factors that caused different levels of anxiety and depression for HCWs in different regions were discussed. It was found that whether you often worry about occupational exposure and daily wear protective clothing time (hours) exerted significant independent effects for anxiety, and participation in epidemic work (days) and daily wear protective clothing time (hours) exerted significant independent effects for depression. Sex, age, and whether worried about family's cross-infection had no effect on the joint analysis. Fear of occupational exposure is a direct cause of anxiety. This not only increases their own risk of infection, but also threatens the health of their families. Wearing protective clothing for a long time daily reflects the high work intensity of HCWs, as well as an increased risk of infection, which not only causes anxiety but also induces depression. It is suggested that HCWs should not work too many hours a day (18, 26). The results also indicate that depression is directly related to long-term participation in epidemic work. In our survey, 86.57% of HCWs in Wuhan, 89.37% of HCWs in other cities in Hubei except Wuhan, and 71.22% of HCWs in other provinces in China except Hubei were worried about their family's cross-infection because of them. Given the high infectivity of the virus and its transmission through respiratory droplets and close contact, fear of inadvertently endangering members of family and loved ones was a widespread concern among HCWs (8). The HCWs were torn between their own responsibilities and this concern. Although the work of HCWs is high-risk, fortunately, we find that 62.5% of HCWs are proud of their work.

The study's discussion of causation is limited by its cross-sectional nature. A further methodological limitation is the possible social expectation bias because of the use of self-report to assess psychiatric morbidity, some people may be inclined to underreport their psychopathology to avoid discrimination. We also cannot rule out the possibility that people with severe anxiety or depression was under-represented. Pre-COVID-19 anxiety and depression were not collected for HCWs and non-HCWs, so it was not possible to compare mental states before and after the outbreak. Finally, all residents in China are quarantined at home, so generalizations of our findings are limited, it may not apply to other countries. It would be meaningful to study the mental health status of HCWs and non-HCWs in different geographic regions at the beginning of the epidemic, the peak period, the remission period, and longer afterwards. Longitudinal studies with larger sample size are encouraged in future studies to conduct a more comprehensive assessment of this issue.

Despite these limitations, it is clear that HCWs, especially in Wuhan, have serious mental health problems. It is necessary to take relevant measures to treat or manage public anxiety and depression, for it may have a lasting effect after the epidemic ends (14). What's more, improving the psychological health of HCWs is of great significance for maintaining their physical health, keeping higher efficiency and working state, and controlling the epidemic (35, 36). The Chinese government has made some policies to address these psychological health problems and pay close attention to the psychological health of HCWs, such as caring for the elderly and children of HCWs to solve their worries, establishing a shift system so that front-line HCWs can take turns to rest, providing a professional psychological counseling platform, opening a public psychological hotline, and setting up psychological intervention teams to conduct psychological intervention and guidance to the masses through social platforms such as WeChat groups, etc.

In summary, when a new infectious disease breaks out, health care workers, especially where the epidemic is more severe, may bear heavier workloads and higher levels of anxiety and depression. Understanding the mental health response of COVID-19 may help prepare for future outbreaks of infectious diseases and improve the efficiency and quality of future crisis interventions.

## Data Availability Statement

The raw data supporting the conclusions of this article will be made available by the authors, without undue reservation.

## Ethics Statement

The studies involving human participants were reviewed and approved by the Institutional Review Board (IRB) of Huazhong University of Science and Technology. Written informed consent from the participants was not required to participate in this study in accordance with the national legislation and the institutional requirements. The purpose and use of this research were explained at the beginning of the online questionnaire. All the participants provided online informed consent before entering the online questionnaire.

## Author Contributions

HC, GL, and FW designed the study. JL, LL, JZ, YH, CZ, and SH collected data. CZ and YH analyzed the data. FW and YJ administered the project. HC and CZ writing – original draft preparation. FW and GL writing – review & editing. YJ and FW funding acquisition. All authors read and approved the final manuscript.

## Conflict of Interest

The authors declare that the research was conducted in the absence of any commercial or financial relationships that could be construed as a potential conflict of interest.
